# A Flexible, Low-Cost Hydroponic Co-Cultivation System for Studying Arbuscular Mycorrhiza Symbiosis

**DOI:** 10.3389/fpls.2020.00063

**Published:** 2020-02-26

**Authors:** Debatosh Das, Salar Torabi, Philipp Chapman, Caroline Gutjahr

**Affiliations:** ^1^ Faculty of Biology, Genetics, LMU Munich, Martinsried, Germany; ^2^ Plant Genetics, TUM School of Life Sciences Weihenstephan, Technical University of Munich (TUM), Freising, Germany

**Keywords:** hydroponics, hairy root transformation, rice, *Lotus japonicus*, arbuscular mycorrhiza

## Abstract

Arbuscular mycorrhiza (AM) is a widespread symbiosis between plant roots and fungi of the *Glomeromycotina*, which improves nutrient uptake by plants. The molecular mechanisms underlying development and function of the symbiosis are subject to increasing research activity. Since AM occurs in the soil, most studies targeting a molecular understanding of AM development and function, use solid substrates for co-cultivating plants and AM fungi. However, for some experiments very clean roots, highly controlled nutrient conditions or applications of defined concentrations of signaling molecules (such as hormones) or other small chemicals (such as synthetic inhibitors or signaling agonists) are needed. To this end, hydroponics has been widely used in research on mechanisms of plant nutrition and some hydroponic systems were developed for AM fungal spore amplification. Here, we present a hydroponics set-up, which can be successfully utilized for experimental root colonization assays. We established a “tip-wick” system based on pipette tips and rock wool wicks for co-cultivation of AM fungi with small model plants such as *Lotus japonicus*. A larger “Falcon-wick” system using Falcon tubes and rockwool wicks was developed for larger model plants such as rice. The hydroponic system can also be employed for growing *L. japonicus* hairy roots after transformation by *Agrobacterium rhizogenes*, thus circumventing the laborious cultivation on agar medium-containing Petri dishes during hairy root development. The tip-wick and Falcon-wick systems are easy to use and can be built with low cost, conventional and reusable lab plastic ware and a simple aquarium pump.

## Introduction

The roots of most land plant species including prominent economic crops are colonized by beneficial soil fungi of the *Glomeromycotina* to form a symbiotic association called arbuscular mycorrhiza (AM) (Smith and Read, 2008). AM fungi (AMF) are obligate biotrophs and depend on sugar and fatty acid supply by the plant host, while plants benefit from improved mineral nutrition, especially with phosphate, better water retention and biotic, as well as abiotic stress tolerance (Smith and Smith, 2011; Roth and Paszkowski, 2017; Keymer and Gutjahr, 2018; Chen et al., 2018). Fossil evidence suggests that AM has evolved since plants conquered land more than 400 Mio years ago (Remy et al., 1994), suggesting highly adapted genetic and metabolic pathways underlying regulation and function of this symbiosis (Delaux et al., 2015).

Root colonization by AM fungi comprises different steps that can be separated by plant mutants (Gutjahr and Parniske, 2013; MacLean et al., 2017; Choi et al., 2018). After an exchange of molecular signals between the root and the germinating fungal spore (Nadal and Paszkowski, 2013) the tips of growing hyphae attach to the root epidermis by so called hyphopodia. Then the hyphae penetrate the root epidermis and colonize the cortex. Fungal intraradical hyphae spread longitudinally in the root and form specialized highly branched tree-shaped structures, the arbuscules, inside cortical cells (Gutjahr and Parniske, 2013; MacLean et al., 2017; Choi et al., 2018). These arbuscules are surrounded by a plant derived peri-arbuscular membrane and the pair of arbuscule and arbuscule-containing cortex cell is thought to represent the main entity for nutrient exchange between fungus and plant (Luginbuehl and Oldroyd, 2017; Keymer and Gutjahr, 2018). At later stages of the symbiosis, some AM fungi (of the Glomeraceae family) also form vesicles, which are filled with lipids and nuclei and increased vesicle formation is often associated with a decline in the number of intact arbuscules (Kobae et al., 2016).

Although several plant genes required for arbuscular mycorrhiza development and function have been identified (Gutjahr and Parniske, 2013; MacLean et al., 2017; Choi et al., 2018), the interplay of their protein products and the molecular mechanisms regulating root colonization and symbiotic processes are poorly understood and are subject of active investigation.

Since field soil is difficult to wash off from roots and since it may contain numerous microorganisms, which influence the plant and each other, most studies aiming at a molecular understanding of AM development and function use inocula of single fungal isolates and make use of specialized substrates such as quartz sand, a mix of sand and vermiculite, sand and loam or sand and calcined clay. These substrates are more loosely attached to the roots and can be more easily washed off than field soil, although it can be still cumbersome to obtain very clean roots. Solid substrates can have a major disadvantage if the impact of chemical compounds such as hormones, inhibitors, signaling agonist or nutrients on AM development and function is to be examined, because chemicals and nutrients can be adsorbed to the surface of substrate particles or washed out, making it difficult to estimate the exact concentration, to which the roots are exposed. Hydroponic systems can be a solution to this problem. In hydroponics, roots are grown in liquid media, in which nutrient and chemical concentrations can be easily controlled. Furthermore, hydroponic culture provides very clean root material and in addition it represents a practical setup for collecting root exudates.

Hydroponic systems have been previously developed for AM symbiosis and most have been used for commercial inoculum production (reviewed in Ijdo et al., 2011). Several of these hydroponic setups involve growing plants in a solid-substrate for pre-colonization of roots by AMF and transfer to a hydroponics system after several weeks of co-culture (Hawkins and George, 1997; Lee and George, 2005; Utkhede, 2006). Such setups are labor intensive because an extra transfer step is required. Furthermore, they are not suitable for controlled time-course experiments (for example for harvesting colonized roots or root exudates at multiple time points after inoculation) because the growth conditions drastically change during the course of the experiment.

With the interest in studying the molecular mechanisms of AM development and function, we established a hydroponic culture system, in which plants are inoculated with fungal spores directly in the system. The system is easy to set up and requires only simple and general lab plasticware, available in molecular biology laboratories, and simple aquarium supplies. All plastic materials can be reused multiple times. For small plants such as the model legume *Lotus japonicus* we use 1 ml pipette tips and a rockwool wick (called “tip-wick”) as a stable support for fungal inoculation and for guiding the inoculated roots into an aerated nutrient solution. For larger plants such as rice we use 50 ml Falcon tubes in the “Falcon-wick” setup. We show that in this system, roots are readily colonized by an AM fungus and express AM marker genes in a similar manner to roots colonized in pot cultures. Moreover, we demonstrate the suitability of the hydroponic system to study the impact of small molecules on root colonization and also to grow *L. japonicus* transgenic hairy roots after *Agrobacterium rhizogenes* transformation, requiring less work input than the classical protocol of growing hairy roots on Petri dishes.

## Materials and Methods

### Set-Up of the Hydroponic Systems

**Figure 1:**
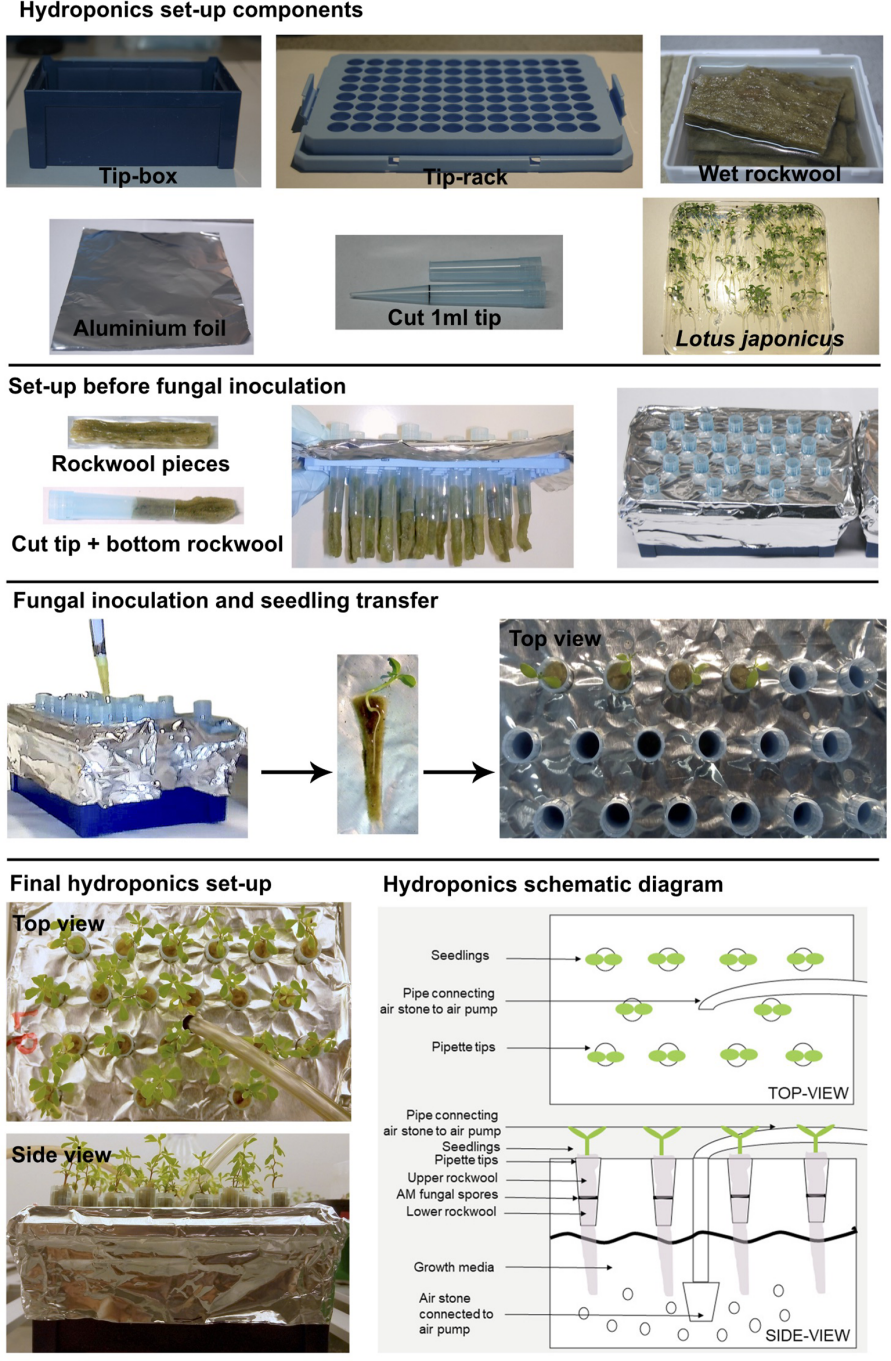
Tip-wick hydroponics. Basic set-up for *Lotus japonicus*.

For the basic tip-wick hydroponic setup, for small plants such as *L. japonicus*, the following components are needed: a tip-box for 200 µl pipette tips (TipOne^®^ Pipette Tip System, StarLab, Germany), tip-rack for 1 ml pipette tips (TipOne^®^ Pipette Tip System, StarLab, Germany), rockwool (Grodan, ROCKWOOL B.V., the Netherlands), which is wetted overnight in autoclaved water, 1 ml pipette-tips (e.g., TipOne^®^ Pipette Tip System, StarLab, Germany), aluminum foil, and *L. japonicus* seedlings (**Figure 1**). All components can be re-used multiple times but should be washed, followed by steam autoclaving or sterilization with bleach and 70% ethanol before re-use. Only the rockwool, has to be used fresh each time.

To assemble the tip-wick system, the rack is first covered with aluminum foil to prevent light exposure of the roots and the medium, and to prevent growth of algae. Then, a bottom rockwool layer is inserted into the cut 1 ml pipette tips, such that half of it hangs to the outside and can act as a wick delivering liquid media to the plant. The tip-wick combinations are then placed into the holes of the 1 ml tip rack by pinching them through the aluminum foil layer. Five hundred spores per tip are then placed on top of each rockwool wick in a 100 µl volume of distilled water or nutrient solution. Then the seedlings are transplanted into the tips (one per pipette tip) by enclosing them into a coat of rockwool (rockwool top layer) and inserting them together with the rock wool into the empty upper half of the tip. We advise users to gently fit the rockwool layers into the tip without twisting or turning it. Twisting and turning would make it excessively tight-fitted to the tip and may create anoxic conditions for the growing roots. We also advise users to put less than 15 *L. japonicus* seedlings per 200 µl tip box, as with higher densities the root systems entangle with each other as the roots grow bigger. The aeration tube is inserted in the middle of the tip-rack (**Figure 1**).

The Falcon-wick hydroponic system for bigger plants such as rice (**Figure 5**) is set up in a black 3-liter bucket with plastic lid (JET 30, JOKEY, USA). Six holes with diameter equal to the outer diameter of 50 ml Falcon tubes are drilled at equal distances in a circle into the lid. For planting and support of fungal spores cut Falcon tubes are stuffed with top and bottom rockwool layers: the fungal spores are pipetted onto the bottom layer and then the top layer gently enclosing a rice plant is inserted into the tube. Subsequently, the tubes are inserted into the holes of the bucket lid. The aeration tube is installed through a hole drilled into the side of the bucket above the water level (**Figure 5**). For aeration of both the tip-wick and the Falcon-wick system, a commercial membrane aquarium pump (for example Sera Air 550 R Plus, Germany) is connected with a tube (4/6 mm inner/outer diameter) to a cylindrically shaped air stone (20x30 mm) for the tip-wick and to a round air stone (80 x 15 mm) for the Falcon-wick system and is inserted into the tip-box or bucket as described above.

The hairy root hydroponics is built in a similar manner to the tip-wick system but with a modified lid and plant support. For the lid, a flat 200 µl tip-rack (epTIPS, Eppendorf, USA) is used (**Figure 4**), from which the two side-clips are removed such that it fits deeper into the container and lines up almost precisely with the height of the box. This is needed to ensure sufficient floating of the plants in liquid medium. Cuttings of the seedlings are surrounded by a small piece of pre-wetted rockwool, a cylinder-shaped piece of sponge or a cigarette filter (Filter Tips extra small, Swan, UK) with a recess, and placed into a hole of the inlay. Directly after placing the seedlings into the tip box, they are covered with the lid of the tip box to avoid drying. If the planting was done directly after transformation, the box is placed for 2 days in darkness. After these two days, the plants are placed into a 16/8 h day/night cycle; the lid of the tip box is removed and the box is placed into a growth tray and covered with the transparent lid of the tray to avoid drying of the shoots. After one to 2 weeks, when the seedlings start to develop roots and are adapted to the environment, the lid can be removed gradually or can remain throughout the experiment if the plants are sensitive. In this case, the lid is placed slightly shifted on the tray to allow some ventilation. After the co-cultivation step of 5 days in hydroponics or on Petri dishes, the nutrient solution is changed to a cefotaxime (330 µg/ml) containing solution and aerated with an air stone connected to an aquarium pump. The nutrient solution with cefotaxime is exchanged weekly. After 2 to 3 weeks the plants are screened for successful transformation and can be used for inoculation in pots with AMF or for other types of experiments. A starvation step before inoculation is not necessary because the phosphate concentration in half-Hoagland nutrient solution can be set to AM compatible levels. Moreover, since the plants are already adapted to the environment in the climate chamber, they do not need to be covered and are less stressed in comparison to the classical protocol where they are planted directly from the closed Petri dish to the open pots.

### Plant Material and Growth Conditions

Seeds of *L. japonicus* ecotype Gifu B-129 and *Oryza sativa* ssp. *japonica* cv. Nipponbare were sterilized using a solution of 0.1% sodium dodecyl sulfate (SDS) and 5% Bleach and germinated on a 1% water-agar plate for 1–2 weeks before the plants were transplanted to the hydroponics set-up with 500 spores of *Rhizophagus irregularis* (strain DAOM 197198) (Agronutrition, Toulouse, France) or to pots filled with washed and autoclaved quartz-sand and inoculated with 500 spores of *R. irregularis* as described (Pimprikar et al., 2016). Plants were grown in a 16/8 h long day photoperiod with 24/22°C temperature cycles and 60% air humidity. The light intensity was 180 µM/m^2^*s (Sylvania LUXLINE PLUS, T5, 39W, light color 830) for most experiments and 150 µM/m^2^*s (Polyklima LEDs true daylight dual, with red channel at position 6 and blue channel at position 1) for the experiment shown in **Figure S1**. Half-Hoagland solution with 2.5 µM phosphate was used as growth medium for both tip-wick and Falcon-wick hydroponics with *L. japonicus* and rice, respectively and for *L. japonicus* pot experiments. For hairy root hydroponics Half-Hoagland solution with 25 µM phosphate was used (Hoagland and Arnon, 1950).

### Gibberellin and Phosphate Treatment

GA_3_ (Sigma-Aldrich, USA) was used to prepare a 50 mM stock solution in absolute ethanol. GA_3_ was added directly to the growth media to a final concentration of 1 µM and the control was supplemented with equal amounts of solvent (0.002% ethanol, v/v). For testing the effect of different phosphate concentrations the Half-Hoagland solution was supplemented with 2.5 or 2,500 µM potassium phosphate. The concentration of potassium in the low phosphate medium was adjusted to 2,500 µM using potassium chloride.

### Quantification of Root Colonization by *Rhizophagus irregularis*

Roots were harvested from hydroponics and stained with ink and vinegar (Vierheilig et al., 1998). Ink-stained roots were cut into 1 cm pieces and observed under a microscope at 200X magnification to score fungal structures using the magnified gridline-intersect method (McGonigle et al., 1990). Percent colonization is presented as: (number of intersections with fungal structures X 100)/total number of intersections.

### Quantification of Transcript Accumulation

Harvested roots were snap frozen in liquid nitrogen and ground in liquid nitrogen. RNA was isolated from the tissue powder using the Spectrum™ Plant Total RNA Kit according to manufacturer's instructions (Sigma-Aldrich, USA). Subsequently, the RNA was treated with DNAse I-Amplification Grade (Sigma-Aldrich, USA), followed by verification of sufficient removal of genomic DNA contamination by PCR using primers exclusively targeting genomic DNA. The quality of extracted RNA was checked by running aliquots of each sample on an agarose gel and quantified using a NanoDrop™ ND-1000 Spectrophotometer. One microgram of RNA for each sample was used for cDNA synthesis with SuperScript^®^ III First-Strand Synthesis System (Thermo-Scientific). Primers for *L. japonicus SbtM1, BCP1, PT4,* and *UBIQUITIN* and rice *PT11*, *ARK* (*AM14*), and *CYCLOPHILLIN2* were previously described (Gutjahr et al., 2008; Pimprikar et al., 2016). Quantitative real-time RT-PCR was carried out using mi-real-time EvaGreen^®^ Master Mix (Metabion, Martinsried, Germany), on a QuantStudio 3 Real-Time PCR system (Applied Biosystems, USA). Data were extracted using QuantStudio 3 Real-Time PCR Data Analysis Software and analyzed for normalized expression following the 2^−∆∆CT^ method (Livak and Schmittgen, 2001). The housekeeping genes *L. japonicus UBIQUITIN* and rice *CYCLOPHILLIN2* were used for normalization of gene expression values in the respective species.

### Fresh Weight Quantification

Fresh weight of *L. japonicus* roots and rice roots and shoots were measured at 4 wpi and 7 wpi, respectively by weighing on an analytical balance.

### Hairy Root Transformation

Hairy root transformation was conducted according to Okamoto et al. (2013). In short, hypocotyls of *L. japonicus* seedlings were dipped in *A. rhizogenes* AR1193 culture transformed with a plasmid carrying an mCherry marker driven by a *UBIQUITIN* promoter (called EV in Pimprikar et al., 2016). Before transformation, the seedlings were germinated for 3–4 days in darkness and for 2–3 days in 16/8 h light/dark cycles on 0.8% water agar plates. For infection, the seedlings were cut in the middle of the hypocotyl on a filter paper with *A. rhizogenes* suspension (containing the DNA construct). After transformation, the seedlings and bacteria were co-cultivated for 2 days in darkness and 3 days in 16/8 h light/dark cycles on Petri dishes containing Gamborg's B5 medium (Duchefa) without sucrose, to avoid overgrowth of the agrobacteria. Then they were transferred to Petri dishes containing Gamborg's B5 medium (Duchefa), 300 µg/ml cefotaxime, and 1% sucrose to inhibit bacterial growth and to develop hairy roots. For regeneration of hairy roots in hydroponics, the seedlings were transferred directly after the transformation or after the co-cultivation step to the hydroponic system containing half Hoagland solution without sucrose. After the co-cultivation step the growth media was supplemented with 300 µg/ml cefotaxime to inhibit bacterial growth. After 2–3 weeks, the root systems were screened for successfully transformed roots using the mCherry fluorescent transformation marker as described before (Pimprikar et al., 2016). The transformation efficiency was determined by calculating the percentage of plants with transformed roots among the total number of *A. rhizogenes* AR1193 infected plants.

### Statistical Analysis and Data Display

Graphs were plotted and statistical tests were performed using Prism 6 software (GraphPad, USA).

## Results and Discussion

### Tip-Wick Hydroponic Culture Is Suitable to Study Arbuscular Mycorrhiza Development

**Figure 2:**
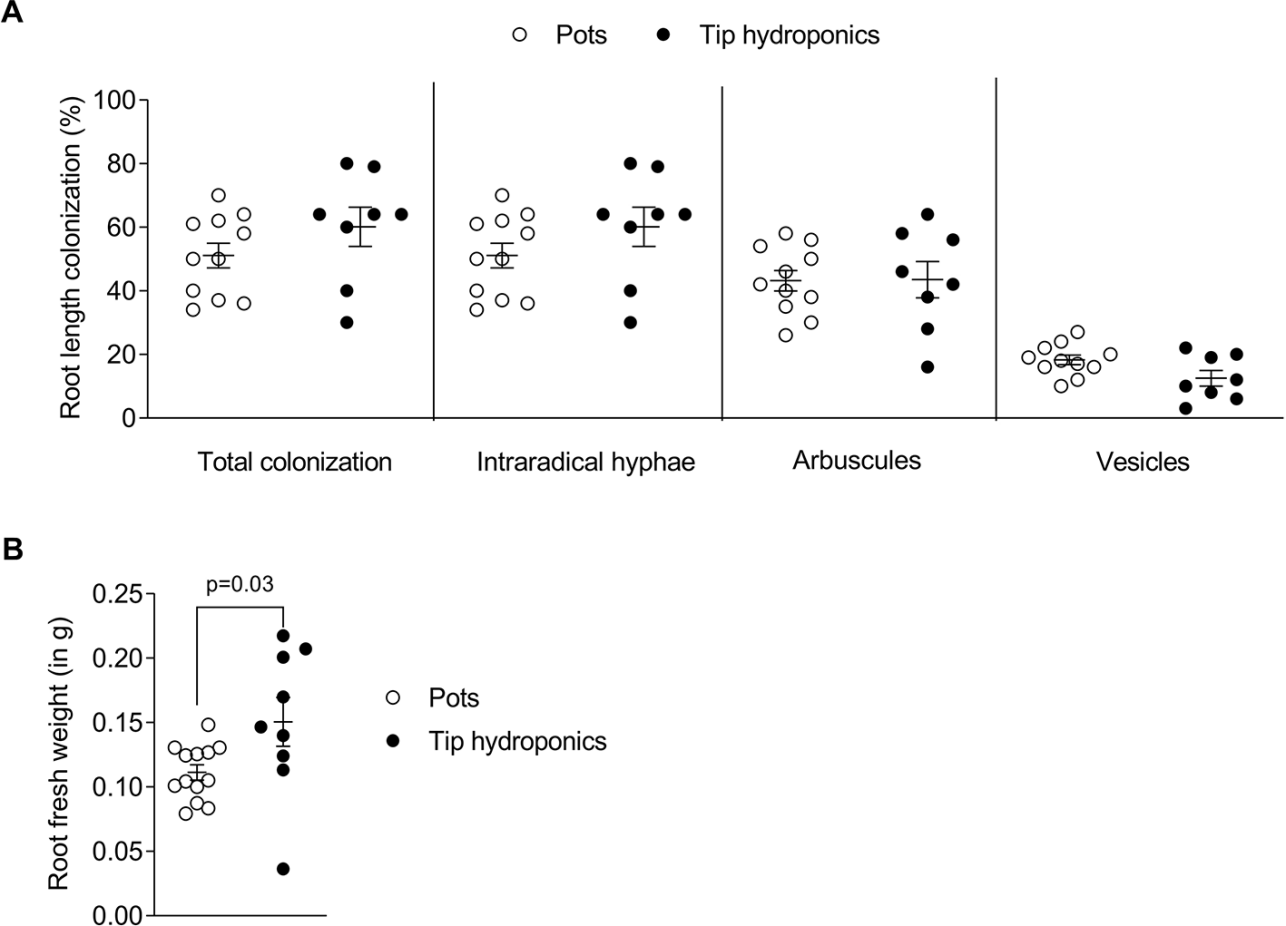
Root length colonization after growth in sand-filled pots *vs*. tip-wick hydroponics. Comparison of **(A)** percent colonization of *Lotus japonicus* roots with *Rhizophagus irregularis* and **(B)** *L. japonicus* root fresh weight in sand-filled pot *vs*. tip-wick hydroponics at 4 weeks post-inoculation (wpi). Mann-Whitney tests were used for statistical comparison (n = 8–15 separate plants from one tip-box or two independent pots).

To understand whether arbuscular mycorrhiza can be studied using a simple hydroponic culture setup we established the tip-wick hydroponics with *L. japonicus* and the AM fungus *R. irregularis* as described in *Materials and Methods*. We compared root growth and colonization by *R. irregularis* between plants grown in hydroponics and in commonly used pots with sand as growth substrate. Due to evaporation, the tip-wick system required around 200 ml of nutrient solution per week for 12–15 plants, while pots were supplemented with 30 ml of media three times per week for 6–9 plants per pot. No significant difference in total root colonization was observed between plants grown in tip-wick hydroponics or sand pots (**Figure 2A**). However, the plants grown in hydroponics had on average a 20% larger root biomass (**Figure 2B**).

**Figure 3:**
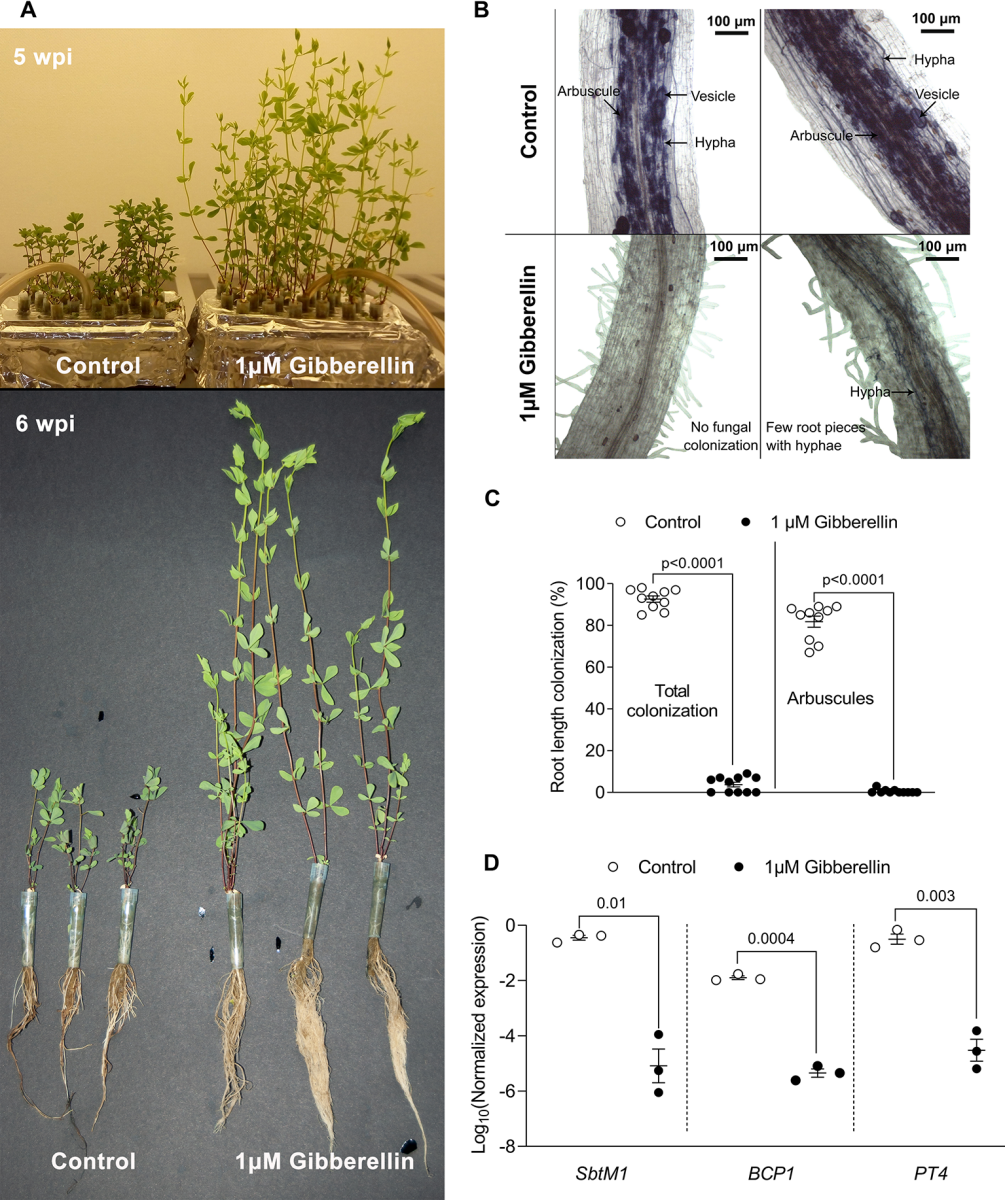
Pharmacological assay in tip-wick hydroponics. **(A)** Effect of 1 µM gibberellin treatment on *Lotus japonicus* growth in tip-wick hydroponics; **(B)** 1 μM gibberellin suppresses root colonization by *Rhizophagus irregularis*. The fungus inside the roots was stained with acid ink. **(C)** Effect of 1 μM gibberellin on percent root length colonization by *R. irregularis* (statistical analysis: Mann-Whitney test, n = 10 separate plants in one tip-box). **(D)** Effect of 1 μM gibberellin on AM marker gene expression (statistics: t-test with Welch's correction, n = 3 consisting of a pool of two root systems each, with the six plants grown in one tip-box). For all analyses, plants were harvested at 6 wpi.

Based on the set up of the tip-wick hydroponic system, AM is assumed to develop first in the rockwool layer inside the tip and the fungus should then grow toward the part of the roots, which float free in the nutrient solution. The speed, with which the whole root length is colonized may depend on the oxygen conditions inside the solution and therefore, possibly on the position of a plant relative to the air stone. To examine this, we recorded the spatiotemporal development of AM inside *L. japonicus* roots grown at two different distances to the air stone in the hydroponic setup (**Figure S1A**). To this end we shifted the air stone to the side and harvested the root from positions close to and far from the air stone at 5, 6, and 7 weeks post-inoculation (wpi). Furthermore, we separated the part of the root system growing inside the tip rockwool (tip-roots) from the roots growing outside of the tip and the rockwool (free-roots) (**Figure S1A**). As expected, the colonization inside the tip proceeded faster and reached a plateau at 6 wpi, whereas the colonization in the free roots lagged behind (**Figure S1B**). However, apart from hyphae travelling inside the root, the free roots could also be colonized from the outside from extraradical mycelium in the liquid, as we detected several successful hyphopodia on free roots (**Figure S1C**). The position relative to the air stone also had an effect on colonization, with roots close to the airstone being slightly more strongly colonized than those far from the airstone. However, inside the tip this effect was only significant for arbuscules at 5 wpi and for vesicles at 7 wpi. The strongest effect of the vicinity to the air stone was observed in the free roots at 7 wpi, since between 5 and 7 wpi colonization hardly increased in this part of the root systems (**Figure S1B**). This shows the importance of aeration for efficient colonization of especially the roots growing outside of the rockwool. Thus, the distance to the air stone should be considered when setting up AM experiments in hydroponics. If root length colonization is to be precisely compared across several containers, roots at same positions relative to the air stone should be directly compared. However, for treatments with strong effects, the impact of vicinity to the airstone does not appear to be critical (see **Figure 3**).

### Gibberellin Treatment Suppresses Root Colonization in Hydroponic Culture

Gibberellins (GAs) are important plant hormones and well-known regulators of plant growth (Tanimoto, 2005). GA also plays a role in AM symbiosis (Floss et al., 2013) and application of active GA (GA_3_) inhibits root colonization by AM fungi in pot grown *L. japonicus*, *Medicago truncatula*, pea, and rice (Floss et al., 2013; Takeda et al., 2015; Pimprikar et al., 2016; Yu et al., 2014; El Ghachtouli et al., 1996). To examine whether tip-wick hydroponics can be used for studying the impact of exogenously applied chemicals on AM symbiosis, we ran a proof of concept experiment and treated *L. japonicus* seedlings in this system with 1 µM GA_3_ or solvent as a control (without considering the position of plants relative to the air stone). As expected, GA_3_ stimulated the growth of *L. japonicus* and we observed a larger root systems and elongated shoots (**Figure 3A**). Simultaneously GA_3_ strongly inhibited root colonization by *R. irregularis* (**Figures 3B, C**). The inhibition of root colonization by GA_3_ was also reflected at the transcriptional level as previously described (Pimprikar et al., 2016): transcripts of the well-established AM marker genes *SbtM1*, *PT4*, and *BCP1* accumulated to high levels in colonized hydroponically growth roots, but were not activated in GA_3_-treated roots in spite of inoculation with *R. irregularis* (**Figure 3D**). High phosphate supply is known to trigger the plant to suppress AM development (Breuillin et al., 2010; Balzergue et al., 2011). We examined whether this was also possible in hydroponics. Indeed, 2500 µM phosphate in the liquid medium lead to a suppression of root length colonization by approximately 40% as compared to 2.5 µM phosphate in the medium (**Figure S2**). Thus, we demonstrate that tip-wick hydroponics can be successfully used for pharmacological or nutrient assays targeted to understand the molecular functioning of AM symbiosis.

### Growth of *Lotus japonicus* Hairy Roots in Hydroponic Culture

**Figure 4:**
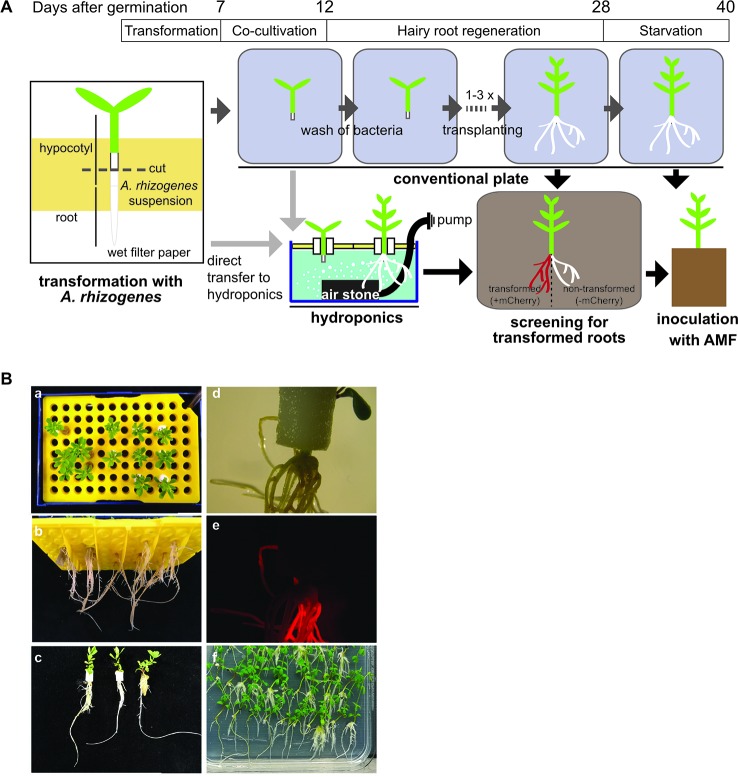
Generation of hairy roots in tip hydroponics. **(A)** Schematic representation of hairy root growth using tip-wick hydroponics. **(B)** Images of mCherry transformed hairy roots of *Lotus japonicus* at 4 weeks post-transformation: (a–c) top, bottom and side view of seedlings grown in hydroponics. Scale bar: 20 mm; (d, e) bright field and fluorescence image of hairy roots generated in hydroponics expressing mCherry as a transformation marker, scale bar: 5 mm; 6. (f) Hairy roots of the same transformation experiment cultivated in square Petri dishes.

In *L. japonicus* the generation of stable transgenic lines takes about 1 year. Therefore, hairy root transformation is a commonly used technique in root symbiosis research on model legumes to generate transgenic roots for experimental use (e.g., to study promoter activity with reporter constructs, or to express tagged proteins) in a shorter period of time (Boisson-Dernièr et al., 2001). After dipping seedling hypocotyls into *A. rhizogenes* solution it takes 2 weeks until hairy roots of 2–5 cm develop (Okamoto et al., 2013). Depending on the purpose of the experiment or the plant genotype, the time to grow hairy roots with a usable size can take even longer. Hairy root cultures of *L. japonicus* require intense care because the plants need to be frequently moved to fresh medium with antibiotics to avoid regrowth of agrobacteria. Before inoculation with AM fungi, all transformed plants need to have a well-developed root system and additionally, the plants need to be starved on low phosphate containing medium for two more weeks before planting them into the pot together with the AM fungus. This means that even with optimal hairy root development, the tissue culture time after transformation can take 4 weeks and more and the plants need to be shifted up to four times to new Petri dishes, which consumes time, plastic ware, and chemicals. We found that, if tissue culture grade sterility of the generated hairy roots is not necessary, the tissue culture steps after transformation can be avoided by growing the hairy roots in hydroponic culture (**Figure 4**).

Directly after transformation, the seedlings can be transferred to the hydroponic system for growth of hairy roots without a decrease in transformation efficiency and with a minimal impact on the survival rate, as compared to a later transfer during root regeneration (**Table 1**). This has the advantage that the transformation does not require tissue culture conditions (clean bench) but can be performed on a clean laboratory bench. However, if many lines are transformed and the work-intensive transformation process shall be split from the transfer to the hydroponic culture among different days, we recommend to perform the transformation and the co-cultivation step under tissue culture conditions using Petri dishes at the clean bench and to transfer to hydroponics the 5 days of co-culture with *A. rhizogenes*.

**Table 1:** *Lotus japonicus* hairy root transformation efficiency and plant survival after growth in hydroponic culture.

Transformation	Transfer to hydroponics after	Plant survival in hydroponics [%]	Transformation efficiency after growth in hydroponics [%]	Transformation efficiency after growth on Petri dishes [%]
1	Dipping	70	80	75
2	Dipping	75	60	45
3	Co-cultivation	80	50	55
4	Co-cultivation	85	100	60

Out of four independent transformations with 50 seedlings each, 20 seedlings per experiment were transferred to hydroponics directly after dipping into *Agrobacterium rhizogenes* solution and 20 seedlings after the 5 days of co-cultivation with *A. rhizogenes*. Transformation efficiency and plant survival were compared to the classical hairy root transformation method with root regeneration on Petri dishes.

### Falcon-Wick Hydroponics for Colonization of Rice Roots by Arbuscular Mycorrhiza Fungi

**Figure 5:**
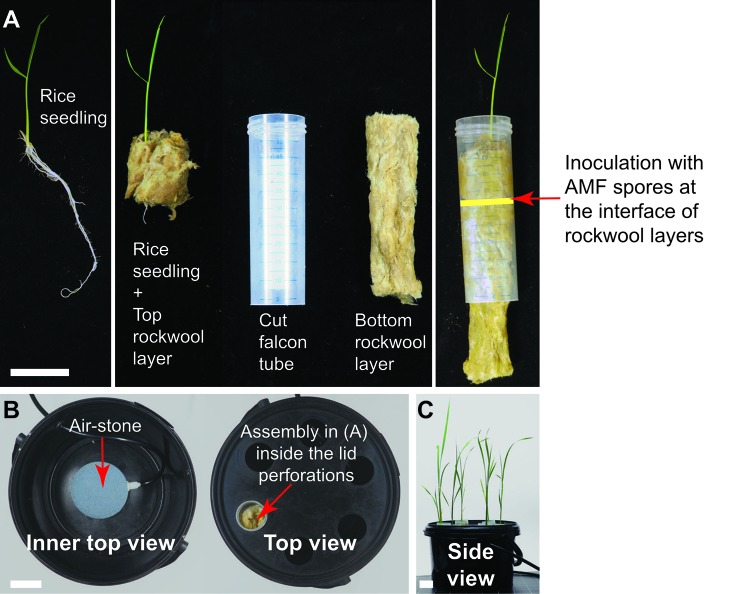
Falcon-wick hydroponics for arbuscular mycorrhiza (AM) of bigger plants such as rice. **(A)** Twelve-day old rice seedling prepared for inoculation with AM fungi (AMF) in hydroponics. Arrow indicates the interface of the two rockwool layers where the AMF spores are placed. **(B)** Top view of the hydroponics set-up in the black bucket. **(C)** Rice growing in hydroponics at 3 wpi. All scale bars: 3 cm.

We also developed a Falcon-wick hydroponics system for larger AM model plants. We choose the important crop and model plant rice, because it currently represents the prime monocots model for studying molecular mechanisms of mycorrhizal colonization (Nakagawa and Imaizumi-Anraku, 2015). We grew rice in a bigger hydroponics system using Falcon tubes instead of pipette tips as a support (Falcon-wick system, **Figure 5**).

**Figure 6:**
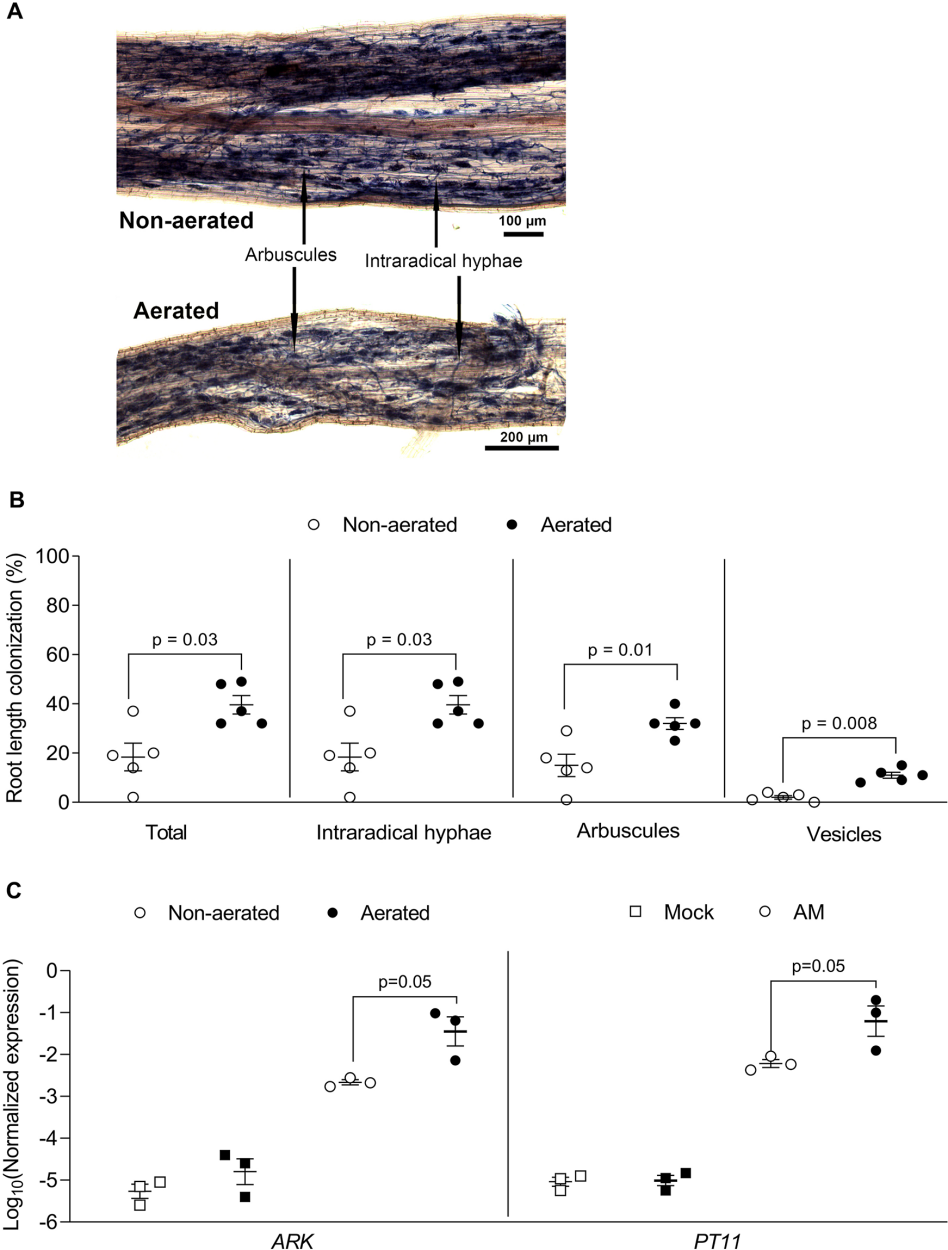
Rice root colonization with *Rhizophagus irregularis* in falcon-wick hydroponics. Rice plants were inoculated with *R. irregularis* and grown in hydroponics for 7 weeks before harvest and analysis. **(A)** Representative images of rice roots colonized with *R. irregularis* and grown in non-aerated and aerated hydroponics. **(B)** Percent root length colonization for different arbuscular mycorrhiza fungi (AMF) structures in rice roots grown in non-aerated and aerated hydroponics (statistics: Mann-Whitney test, n = 5 separate plants grown in one bucket). **(C)** Effect of aeration on AM marker gene expression as determined by quantitative PCR (qPCR). The expression value was of *ARK* and *PT11* was normalized to the expression value of the constitutively expressed gene *CYCLOPHILLIN2* (statistics: Mann-Whitney test, n = 3 independent root systems per treatment).

Rice was grown with and without aeration in hydroponics to test, if aeration promotes AM colonization, as it has been observed that rice displays a low AM colonization level in submerged paddy fields (Solaiman and Hirata, 1995; Vallino et al., 2014). In both conditions, roots and shoots grew a similar biomass (**Figure S3**). *R. irregularis* colonized the roots under both conditions without being morphologically affected (**Figure 6A**). Root length colonization under aerated conditions reached about 40% at 7 wpi, similar to colonization levels observed in sand culture in ConeTainers^®^ (Gutjahr et al., 2008). Under non-aerated conditions, root length colonization was lower and reached about 20% (**Figures 6B, C**). We also examined whether the AM-marker genes *PT11* and *ARK* (Gutjahr et al., 2008; Yang et al., 2012; Roth et al., 2018) are normally expressed in hydroponics. Their transcripts accumulated to high levels in colonized roots as compared to non-colonized roots and their transcript accumulation was slightly affected by aeration (**Figure 6C**). Thus, colonization of rice roots appears to function normally at a molecular level in hydroponics. However, for efficient root colonization of rice in the Falcon-wick system aeration is recommended.

## Conclusion

Here we show that the tip-wick and Falcon-wick hydroponics are practical and easy-to-use setups to study AM, especially when experiments require precise application of chemical compounds or nutrients or very clean root material. Another advantage of hydroponics is that the root growth can be visually monitored in a non-destructive manner, as compared to pot-based growth, because the roots can be repeatedly taken out of the solution and observed. Thus, if required, root growth can be measured repeatedly, which is not possible in pot culture with solid substrates.

## Data Availability Statement

All datasets generated for this study are included in the article/**Supplementary Material**.

## Author Contributions

DD, ST, and CG designed experiments and wrote the manuscript. DD performed all experiments displayed in the manuscript except hairy root transformation, **Figure 6C** and **Figure S1**. ST had the first idea of setting up hydroponics for AM, established both hydroponics systems for AM, for hairy root transformation and performed hairy root transformation as well as experiments for **Figure 6C**. PC performed the experiment for **Figure S1** with help from ST. DD and ST analyzed data with inputs from CG. CG acquired funding.

## Conflict of Interest

The authors declare that the research was conducted in the absence of any commercial or financial relationships that could be construed as a potential conflict of interest.

## Supplementary Material

Figure S1 Spatiotemporal AM colonization in tip-wick hydroponics. (**A**) Experimental set-up to examine the effect of distance to the air stone (c, close; f, far) and position in the root (tip or free) on AM colonization in *L. japonicus* seedlings. (**B**) *L. japonicus* root length colonization by *R. irregularis* at 5, 6, and 7 wpi inside the tip or in free-floating roots, close (open circles) or far (black circles) from the air stone (statistics: Mann-Whitney test, n = 3 separate plants from the three independent tip-boxes). (**C**) Free-floating root length (left) and number of hyphopodia in free-floating root length (right) indicating that the fungus can re-colonize the root from the outside below the rock wool (n = 3 separate plants from three independent tip-boxes). Indications of centimeters on the y-axis refer to root segments below the rock wool. Less than three open or closed circles indicate that not all root systems were long enough to contribute a segment in the indicated category.

Figure S2 High phosphate mediated inhibition of root colonization in tip-wick hydroponics. Effect of 2.5 μM and 2500 μM phosphate on percent root length colonization in *L. japonicus* by *R. irregularis* at 6 wpi (statistical analysis: Mann-Whitney test, n = 9 separate plants from one tip-box).

Figure S3 Rice growth in co-culture with *R. irregularis* in Falcon-wick hydroponics. (**A**) Rice plants grown in non-aerated (left) or aerated (right) hydroponics. (**B**) Root and shoot fresh weights for rice plants grown in non-aerated and aerated hydroponics (statistics: Mann-Whitney test, n = 5 separate plants from one bucket).
