# Supplementary figures and images for: A Flexible, Low-Cost Hydroponic Co-Cultivation System for Studying Arbuscular Mycorrhiza Symbiosis
Source: Front Plant Sci. 2020 Feb 26;11:63. doi: 10.3389/fpls.2020.00063 (PMC7057232; doi:10.3389/fpls.2020.00063)

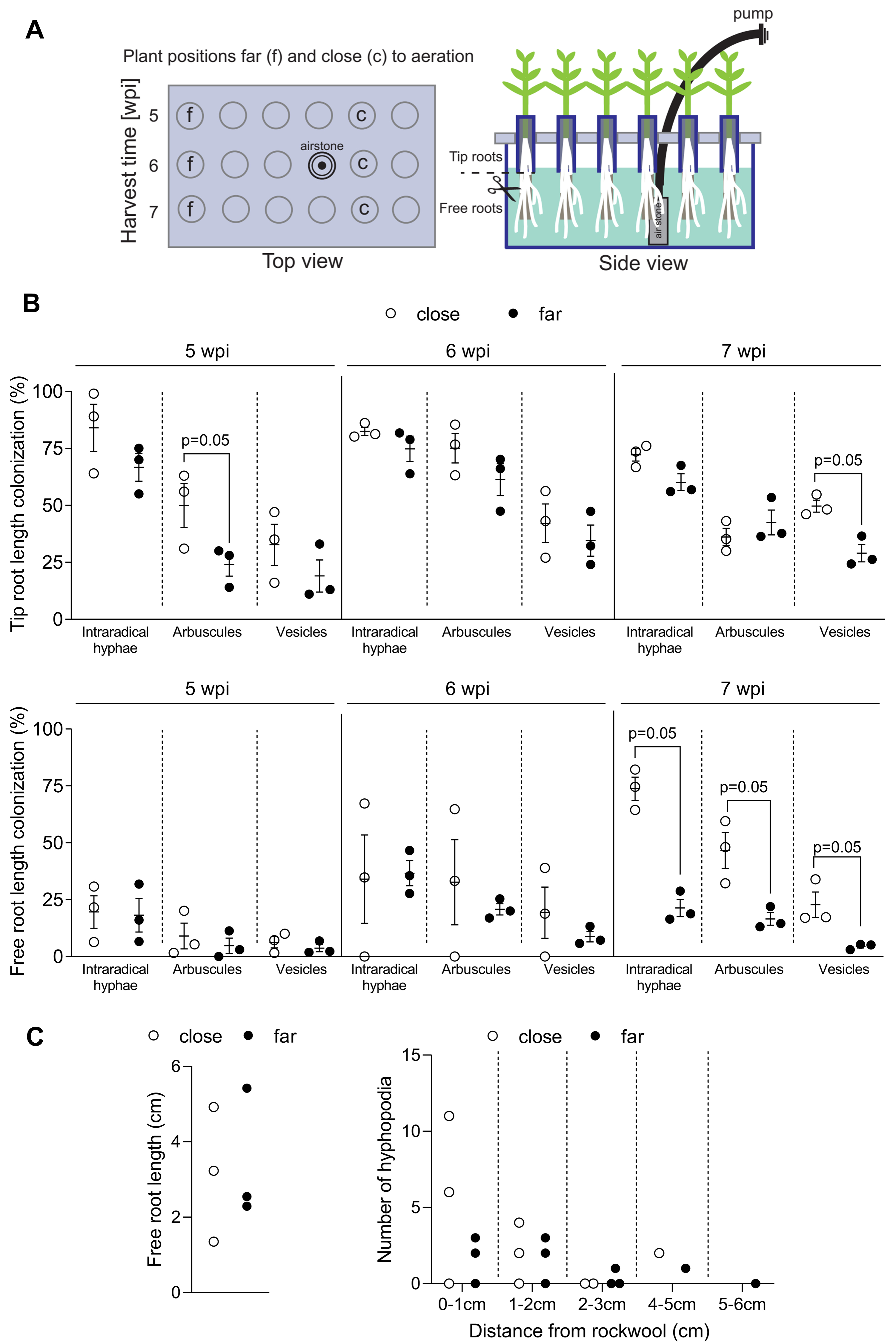

Supplement: Figure S1 — Spatiotemporal AM colonization in tip-wick hydroponics. (A) Experimental set-up to examine the effect of distance to the air stone (c, close; f, far) and position in the root (tip or free) on AM colonization in L. japonicus seedlings. (B) L. japonicus root length colonization by R. irregularis at 5, 6, and 7 wpi inside the tip or in free-floating roots, close (open circles) or far (black circles) from the air stone (statistics: Mann-Whitney test, n = 3 separate plants from the three independent tip-boxes). (C) Free-floating root length (left) and number of hyphopodia in free-floating root length (right) indicating that the fungus can re-colonize the root from the outside below the rock wool (n = 3 separate plants from three independent tip-boxes). Indications of centimeters on the y-axis refer to root segments below the rock wool. Less than three open or closed circles indicate that not all root systems were long enough to contribute a segment in the indicated category. [file Image_1.jpeg]

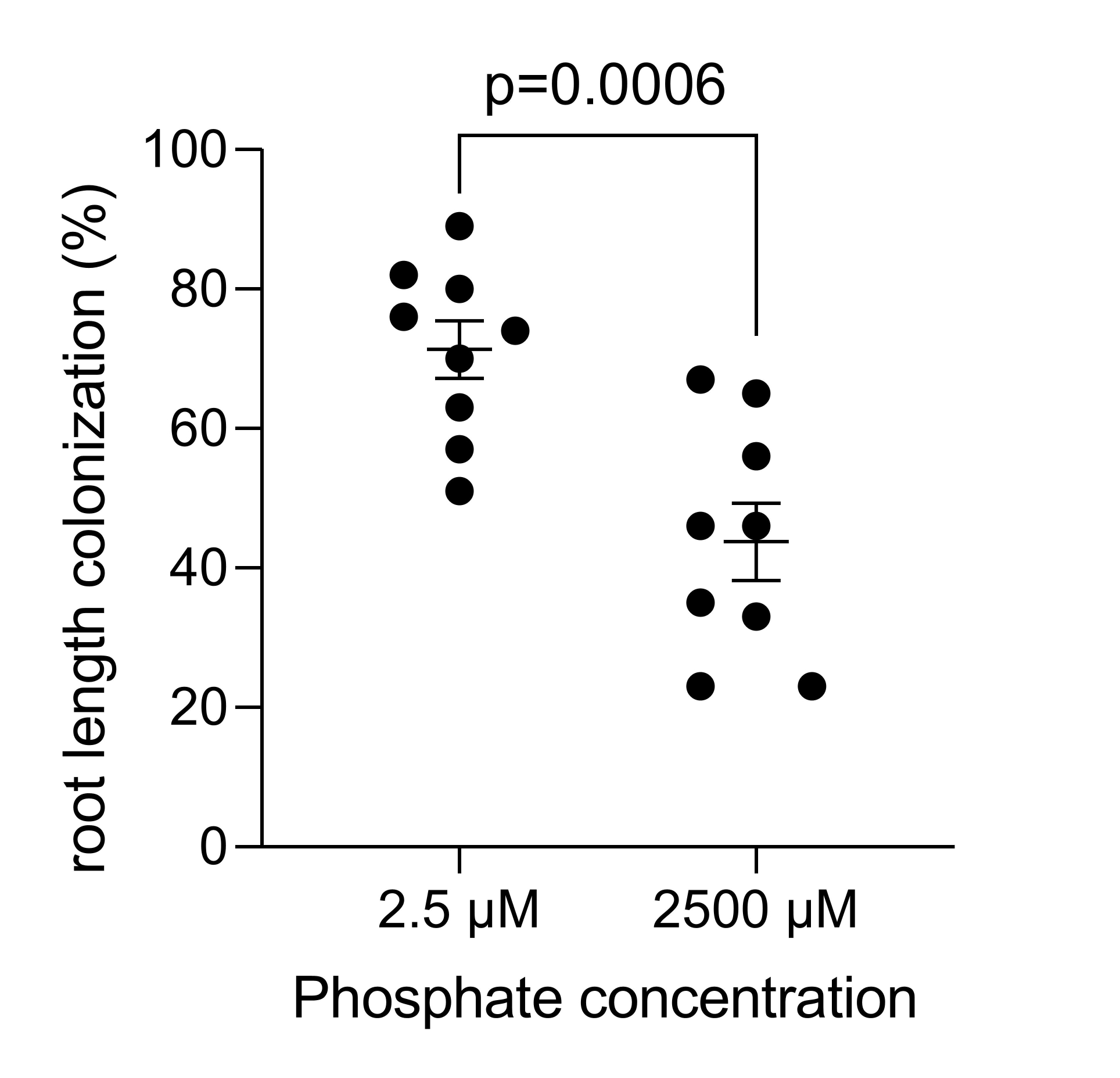

Supplement: Figure S2 — High phosphate mediated inhibition of root colonization in tip-wick hydroponics. Effect of 2.5 μM and 2500 μM phosphate on percent root length colonization in L. japonicus by R. irregularis at 6 wpi (statistical analysis: Mann-Whitney test, n = 9 separate plants from one tip-box). [file Image_2.jpeg]

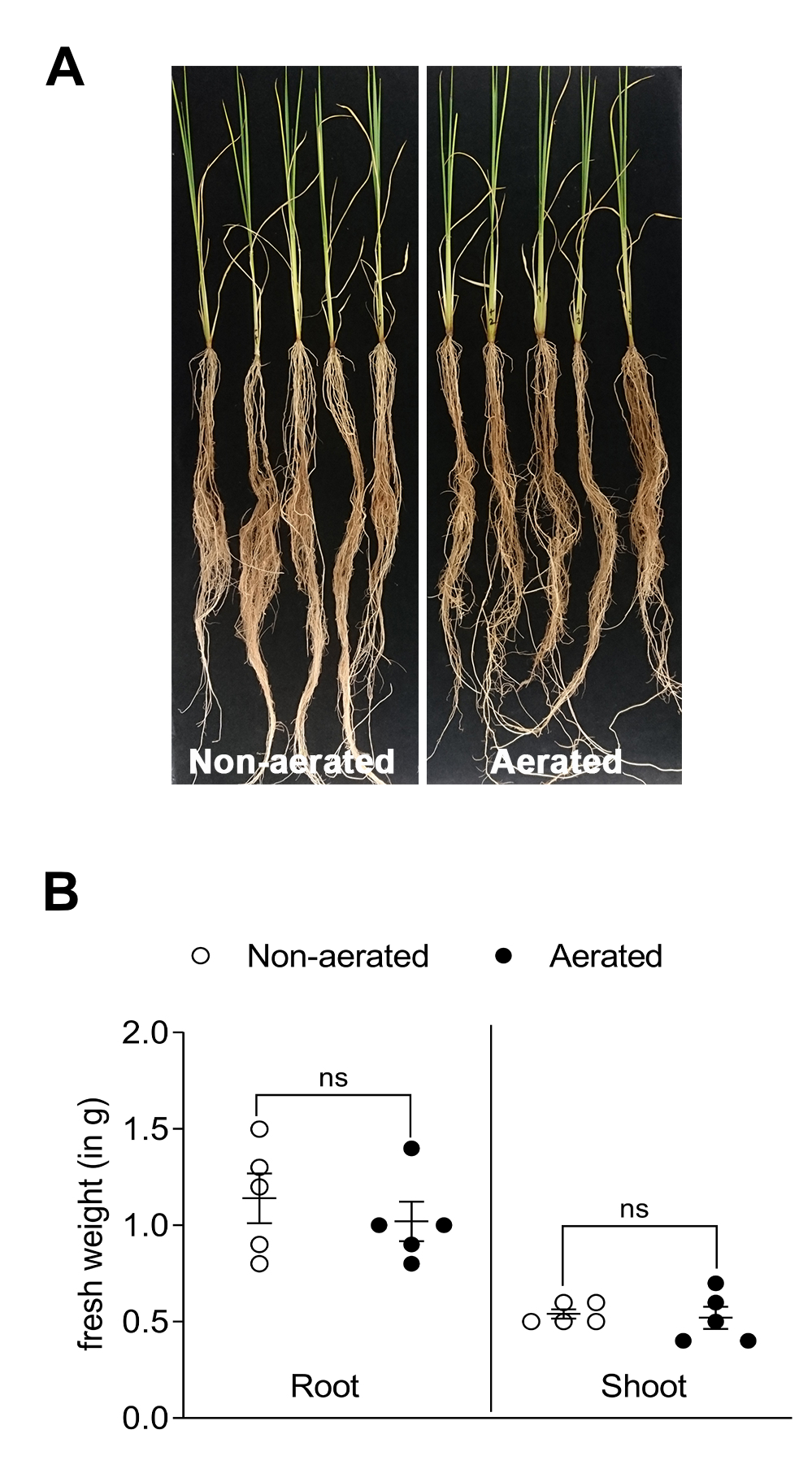

Supplement: Figure S3 — Rice growth in co-culture with R. irregularis in Falcon-wick hydroponics. (A) Rice plants grown in non-aerated (left) or aerated (right) hydroponics. (B) Root and shoot fresh weights for rice plants grown in non-aerated and aerated hydroponics (statistics: Mann-Whitney test, n = 5 separate plants from one bucket). [file Image_3.jpeg]
